# Complex Building Forms Roofed with Transformed Shell Units and Defined by Saddle Surfaces

**DOI:** 10.3390/ma15248942

**Published:** 2022-12-14

**Authors:** Jacek Abramczyk, Katarzyna Chrzanowska

**Affiliations:** Department of Architectural Design and Engineering Graphics, Rzeszow University of Technology, Al. Powstańców Warszawy 12, 35-959 Rzeszów, Poland

**Keywords:** regular systems of shells, parametric polyhedral networks, polygonal networks, division coefficients, corrugated shell roofs, complex substitute material, unconventional building free-forms

## Abstract

A novel method and description of creating diversified complex original building forms roofed with a number of transformed folded shell units developed on the basis of a novel reference polyhedral network and arranged according to a reference surface with the negative Gaussian curvature is presented. For that purpose, specific reference polyhedral networks is are defined as a complex material deliberately composed of many regular tetrahedrons that are arranged regularly to obtain original attractive complex general building forms. The proposed method is a significant extension of the previous method for shaping roof structures with the positive Gaussian curvature and fills existing gaps in current scientific knowledge. The extended method enables the designer to significantly increase the variety of the created complex shell roof forms and plane-walled folded elevation forms of buildings and to define the shapes of their rod structural systems. It allows one to overcome the existing significant geometric and material limitations related to shape transformations of nominally flat rectangular folded steel sheets into different shell forms. The developed extension is based on formation of a set of properly connected tetrahedra as a material determining different (a) inclination of elevation walls to the vertical, and (b) distribution of many individual warped roof shells in accordance with the properties of a regular surface with negative Gaussian curvature. A number of the adopted specific sets of division coefficients (parameters) is used for determining the entire network and its complete tetrahedra. The presented description makes it possible to adopt appropriate assumptions and data and then employ the innovative method to obtain the expected characteristics of the unconventional building form shaped. The presented three different special forms created with the help of the novel method and the appropriately selected diversified values of the division coefficients of pairs of the vertices of a polyhedral reference network, a polygonal eaves network and points of a reference surface confirm the innovative scientific nature of the obtained results. The method has to be computationally aided due to the complexity of mathematical operations and the need to visualize the designed forms.

## 1. Introduction

**Figure 1:**
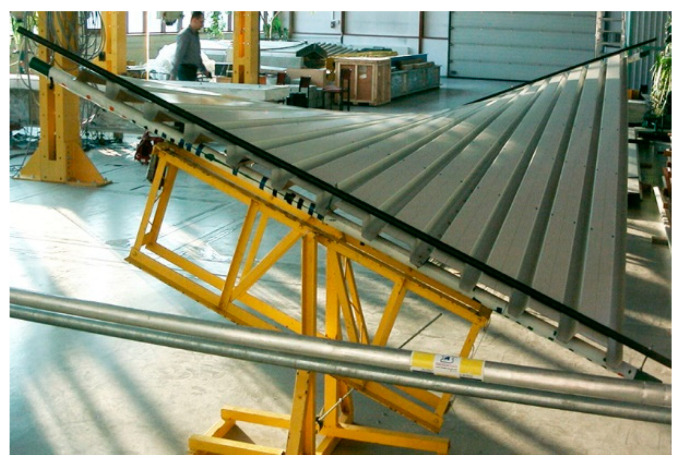
Transformed folded shell sheeting twisted with two skew directrices.

Nominally flat folded rectangular steel sheets are characterized by relatively low transverse bending and longitudinal torsional stiffness [1]. This property enables the designer to exploit their diversified elastic transformations to shape diversified ruled shell forms [2] (Figure 1). However, there are significant geometric and material limitations in the formation of the shells due to a significant influence of these and other stiffnesses on the final shape of the transformed shells [3]. The limitations very often result in significant values of stresses and, for the cases of high transformation degrees, significant values of strains. The intentional spatial shape deformations are called initial transformations [2].

**Figure 2:**
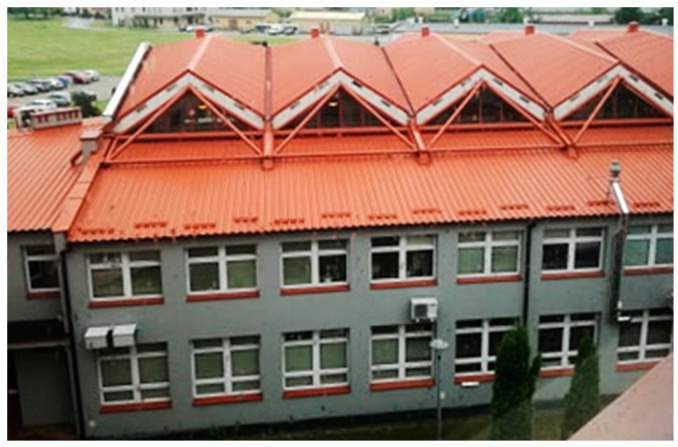
An experimental hall roofed with a regular shell structure.

Further limitations are the straightness of all transformed shell folds and the rectangular shape of the sheets before transformation. On the one hand, these limitations simplify the process of designing and assembling the transformed roof shells. On the other hand, they induce the edge lines of the developed building models to be shaped in the form of various ruled surface sectors [4] each of which is limited by a closed spatial quadrangle with vertex angles close to right [2]. Due to the limited length of complete folded sheets used for roofing, single shell sheeting, being the result of joining the individual sheets with their adjacent longitudinal edges, can cover buildings with a span of up to 15–17 m. Therefore, for medium and long span roofs, edge roof structures composed of a few single folded transformed shells must be accomplished. The simplest way to shape the complex building structures is to determine special sums of many single shells transformed into parabolic-hyperbolic folded sectors, the edge lines of which are closed spatial quadrangles [4,5]. Moreover, all facade wall pieces of the structures have to be flat polygons (Figure 2). In order to obtain a large variety of the designed building forms, the façade walls are inclined to the vertical.

Bearing in mind the above-mentioned assumptions and limitations, a novel polyhedral network called a reference network was developed [6]. The network is used as a geometric material for modeling various unconventional forms of buildings characterized by plane-walled folded facades and edge roof structures composed of many transformed single folded shells. The structure of the layers used in the corrugated sheeting and their temperature affect the mechanical and geometrical properties of these coating segments [7,8].

Each configuration of the network is defined by an appropriate arrangement of many tetrads of vertices defining typical tetrahedral meshes connected by common triangular walls and adequately filling the three-dimensional space. These vertices, planes and side edges of the tetrahedra define the planes and edges of all facade walls of each modeled building. In the above planes, straight segments forming closed spatial quadrangles defining single shell roof are determined. The quadrangles constitute a polygonal network of the roof eaves structure and delimit all individual single roof shells. By varying the geometric properties of the polyhedral reference network and the polygonal network, in particular their subsequent meshes, some variety of building forms can be obtained, including multi-wall folded facades and multi-shell ribbed roofs [9].

## 2. Critical Analysis of the Present Knowledge

Many diversified unconventional building forms and structural system intended for these forms are presented by Abdel and Mungan [10]. The comprehensive classification of different types of a large number of unconventional structural systems is published by Saitoh [11]. Most of these systems can be employed for structural systems supporting complex building forms characterized by folded elevation walls and multi-segment shell structures [9].

Great theoretical possibilities in the field of shaping various ruled forms made up of the transformed folded sheets with an open profiles have resulted in extensive research development in the field of single shell sheeting transformations and creation of multi-segment shell structures [2]. On the basis of the tests and analysis carried out, Bryan and Davis found that some significant material and technological limitations drastically reduce the diversification of the shell forms made up of transformed sheeting to a basic type of shallow hyperbolic paraboloids called hypars [12].

However, the developed novel Reichhart’s method enables one to increase the diversity of shaping the unconventional roof shells and their structures composed of the nominally flat single-layer sheeting transformed into the position of the rigid skew directrices generating folded shell shapes [3].

The results of the tests performed by Abramczyk [13] indicate an important role played by a contraction of each transformed shell sheeting in shaping unconventional forms of buildings roofed with thin-walled folded sheeting transformed elastically into different ruled surfaces [4]. Abramczyk has added the condition related to the central location of the contraction in all effectively transformed folded shells to the Reichhart’s algorithm.

The contraction of a roof shell can be modeled with a line of striction of a ruled surface. Thus, the Reichhart’s method was extended with the boundary condition related to the line of striction. This action should result in a rational shaping of the shell roof forms so that they could be characterized by the relatively high visual attractiveness and the minimum effort [14]. If athe central location of the contraction, halfway along the length of each shell fold, is achieved by means of the arbitrary roof directrices, then the static-strength work of the transformed sheeting is stabilized and rationalized [4]. In this way, a freedom of designing of free-form roof and elevation structures should be achieved [2].

Biswas and Iffland [15] elaborated two trivial systems of many congruent rectilinear shell units distributed over a sphere with the help of bundles of planes, where the complete transformed shells are made of revolved hyperboloids or right hyperbolic paraboloids restricted by spatial straight quadrangles. The proposed method for shaping ribbed structures composed of many congruent shells can be extended to create complex systems of many complete ruled shells separated by sets of planes containing their edge lines.

Prokopska and Abramczyk have carried out some simple systems of reference tetrahedrons to model complete free forms with oblique and folded plane elevations, and roofed with many transformed complete shell sectors [16,17]. The presented building structures are often composed of quarters of hyperbolic paraboloids arranged symmetrically in different configurations. The proposed diversified free form building structures roofed with complex corrugated shells are characterized by medium spans.

For the engineering developments, Abramczyk has proposed that each single shell segment is to be modeled with a sector of a warped surface [2] limited by a closed spatial line composed of four straight segments contained in four planes of a polyhedral reference network [6]. The planes of the reference network are a specific system dividing the designed roof structure into many complete shell segments arranged regularly in the three-dimensional space. In this way, all directrices of the shell sectors can be easily defined in these planes [9].

Pottman has proposed a few comprehensive methods for shaping the systems of planes separating subsequent plane and smooth shell sectors arranged on different regular surfaces [18]. If we want to shape transformed folded shell sheeting by means of the methods, their significant modifications taking into account the geometric and material constraints of the folded sheets must be performed. A few methods carried out by Attard [19], Vlasow [20] or Vasiri [21] can also be implemented to design thin-walled sheeting subjected to large displacements. Samyn has described a method for creating the transformed folded shells made up of aluminum or PVC [22].

**Figure 3:**
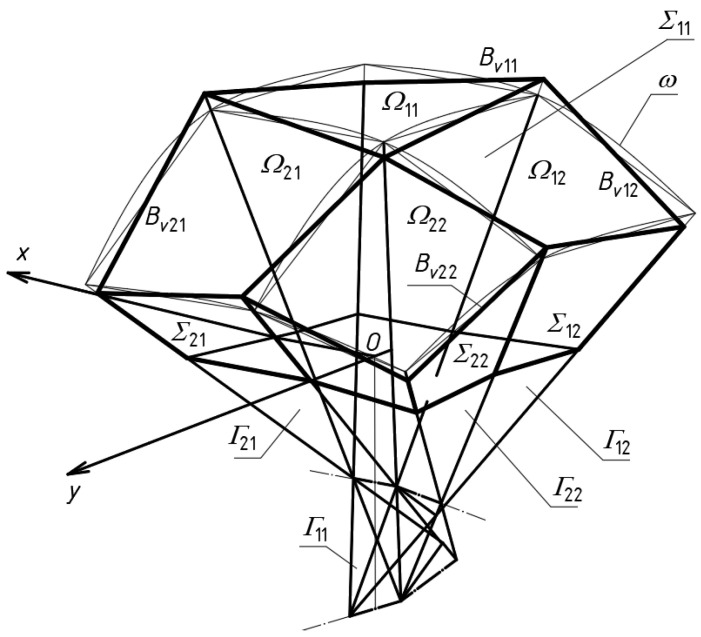
A free-form building structure roofed with a regular continuous shell structure characterized by the positive Gaussian curvature.

The procedure for shaping complex building structures covered with transformed shell roof structures, the general form of which is characterized by a positive Gaussian curvature (Figure 3), has been described by Abramczyk [6] in a fairly accurate manner. This procedure has also been implemented in computer procedures [23]. However, there is no analogous procedure and implementation where the overall form of the shell roof structure complies with the negative Gaussian curvature. This issue is analyzed in detail in this article.

**Figure 4:**
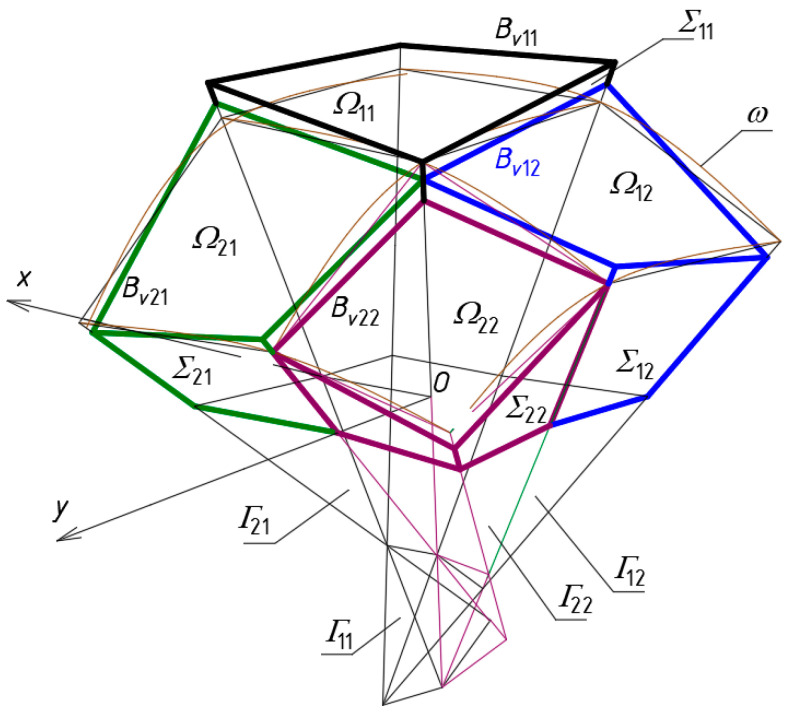
A free-form building structure roofed with a regular discontinuous shell structure characterized by the positive Gaussian curvature.

The analogous universal systems of planes called polyhedral reference networks are utilized in this article. On the basis of these systems, some derivative systems, including polygonal or shell sector networks can be defined to achieve different free-form buildings characterized by complex folded elevation walls and multi-sector shell roof structures (Figure 4) [24].

To increase the attractiveness of the building edge shell structures and the whole urban system, Prokopska has proposed green plant gardens located on the shell roof segments and specific communication routes between the segments [25]. The space around the designed building form, its physical form, urban greenways and cultural patterns of a whole spatial system have to be investigated. Sharma has described [26] the relation appearing between the formation of the urban space and the social experience of the human self. Hasgül [27] presents very important and interesting mathematics-based graph studies of patterns and shapes, thermal based photography and morphology related to the design syntax. Eekhout [28] provides the results of his research in the field of forms taking account of the relationships occurring between the function, structure, internal and external texture, static-strength work and comfort conditions. The systematic morphology leads to the rationalization of each design process.

A very interesting approach to exploit the mechanical properties of new materials making it possible to shape original forms of elevation and roof structures is used by Marin et al. [29], where the classical theory of elasticity developed by Green and Lindsay is extended in terms of the thermo-elasticity theory for dipolar bodies. The proposed novel method is based on a reciprocal theorem and not restrictive boundary conditions [30].

A method for designing complex architectonic forms and engineering rational systems has been developed by Ręebielak [31]. A number of attractive and effective structures adapted to human needs and the built environment has been provided by Tarczewski at al. [32].

The methods for shaping single and complex shell roofs presented in the above literature should be directed towards making each roof from many nominally flat rectangular thin-walled corrugated steel sheets. In order to use these sheets for shell roof covering, shape transformations are required. It is advisable to shape the edge line of each shell segment in the form of a spatial quadrangle with corner angles close to straight and take into account the shape changes and limitations resulting from the shape transformations.

The given references refer to single shells or simple ribbed structures composed of several single shells. The innovative method developed by Abramczyk allows one for a significant increase in the variety of general building forms. However, the method is limited only to structures, where the arrangement of all individual shell segments is consistent with the properties of surfaces characterized by the positive Gaussian curvature (saddle surfaces). In some articles published by the researcher, the possibility of using various structures composed of many single shells distributed on regular surfaces with the negative Gaussian curvature (saddle surfaces) is identified.

However, a comprehensive novel method is presented in the present article and fills the gap in the current knowledge. This area is also important because torsional transformations of a single folded sheet lead to its various shell forms with the negative Gaussian curvature (saddle surfaces). After filling this gap, the authors intend to propose some innovative structural systems supporting the transformed folded shells and extend laboratory tests in the field of thin-walled folded sheeting transformed elastically into various forms of saddle surfaces.

## 3. The Aim

The aim is to present an innovative procedure leading to the creation of some building models characterized by unconventional folded forms of their façades and roofs, where the façade walls are inclined both inside and outside the designed building, and individual roof whose shell sectors are arranged in a three-dimensional space according to the properties of a surface with the negative Gaussian curvature. In order to achieve the above-mentioned goal, a novel algorithm for creating a specific type of the polyhedral reference networks has to be used. The algorithm is implemented into a new procedure for shaping complex building forms in such a way that the created models are located between the vertices of the reference network. However, for the case of the previous building structures with the positive Gaussian curvature, the vertices are positioned on the same side of a reference surface.

The presented procedure allows for arranging the facade and roof elements in relation to the vertices of a reference network using appropriate coefficients expressing some proportions between the distances of the appropriate pairs of the adjacent vertices, and the distances of the characteristic points of the created models from the respective vertices of the reference network. The differentiation of the values of the coefficients adopted as independent variables leads to the differentiation of the obtained building forms. The degree of differentiation of the created models depends on the number of the assumed independent variables. In particular, all the coefficients used may depend on one parameter, which leads to adopting one independent variable and obtaining a single set of building forms with little differentiation.

It is decided that a detailed presentation of the proposed procedure is going to be made using some specific examples. A few different forms obtainable by the procedure are given to observe the possibilities of the procedure. A detailed description of the whole set of the examined diversified complex building forms goes beyond the scope of this article. There are only presented the main relationships governing the position of each mesh *B_vij_* (each shell unit *Ω_ij_*) in the eaves network *B_v_* (the shell structure *Ω*) and three sets of the values of the partition coefficients assigned to the vertices of the same basic reference polyhedral network to analyze three specific types of the general building forms that are different to each other.

## 4. The Method’s Concept

At first, a network *Γ* composed of a number appropriately arranged tetrahedra *Γ_ij_* must be defined. On the basis *Γ*, a model *Σ* of a complex folded building form can be created s as follows (Figure 3). The walls of *Σ* should be included in the planes of *Γ*. The elevation edges of *Σ* should be included in the side edges of *Γ*. The eaves lines of each single shell of the roof structure must be contained in the planes of *Γ*. The vertices of each eaves line must belong to the respective side edges of *Γ*. In the presented procedure, these vertices have to be located between the vertices of *Γ*. 

The horizontal base plane *P_b_* of the whole structure *Σ* must intersect all side edges of *Γ*. The intersecting points should also belong to the side edges and lie between the vertices of *Γ*. The division coefficients of the side edges of *Γ* by the vertices of the base and *Σ* must ensure appropriate positions of these vertices.

**Figure 5:**
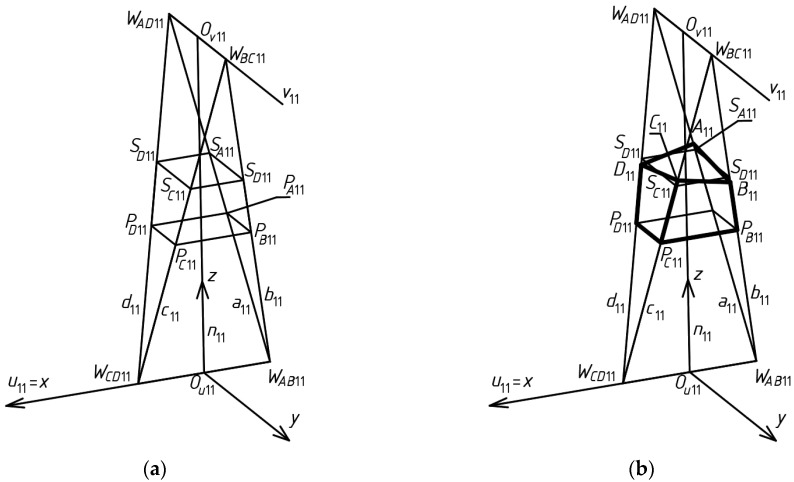
Geometric elements and their denotation used for shaping the first mesh *Γ*_11_ of a polyhedral reference network *Γ*, the first mesh *B_v_*_11_ of a quadrilateral eaves network, and the first mesh of the model *Σ* shaped: (**a**) the essential elements of these networks, (**b**) the obtained first mesh of the model *Σ*.

Construction of *Σ* begins with the determination of the first central tetrahedral mesh *Γ*_11_ of *Γ*. *Γ*_11_ is created on the basis of two arbitrary skew straight lines *u*_11_ and *v*_11_ perpendicular to each other, called the axes of *Γ*_11_ (Figure 5a). An arbitrary distance d*_uv_*_11_ between *u*_11_ and *v*_11_ has to be adopted. It is measured along the straight line *n*_11_ perpendicular to *u*_11_ and *v*_11_ between the points *O_u_*_11_ and *O_v_*_11_ of intersecting *n*_11_ with *u*_11_ and *v*_11_.

In order to determine the positions of four vertices *W_AB_*_11_, *W_CD_*_11_, *W_AD_*_11_ and *W_BC_*_11_ of *Γ*_11_, it is necessary to adopt the distance duv_11_ between the above skew axes, the distance d*_u_*_11_ between *W_BC_*_11_ and *W_AD_*_11_ and the distance d*_v_*_11_ between *W_AB_*_11_ and *W_CD_*_11_. Based on the above assumptions, it is possible to calculate the coordinates of the above vertices in the orthogonal coordinate system [*x*,*y*,*z*] and define four sides of *Γ*_11_ (Figure 5a).

**Figure 6:**
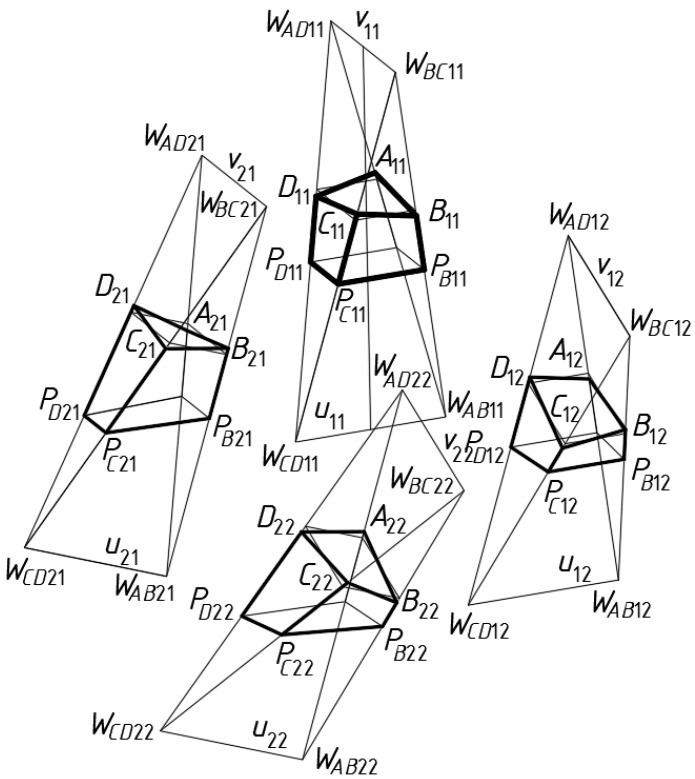
Four subsequent meshes of the designed reference network *Γ*, the eaves network *B_v_*, and the model *Σ*.

A few subsequent meshes are located in orthogonal and diagonal directions of *Γ*_11_ (Figure 6) as follows. For the case of the mesh *Γ*_12_, three vertices *W_AB_*_12_, *W_CD_*_12_ and *W_AD_*_12_ are adopted to be identical to the vertices *W_AB_*_11_, *W_CD_*_11_ and *W_BC_*_11_ of *Γ*_11_, respectively. The axis *u*_12_ of *Γ*_12_ is assumed to be identical to *u*_11_.

**Figure 7:**
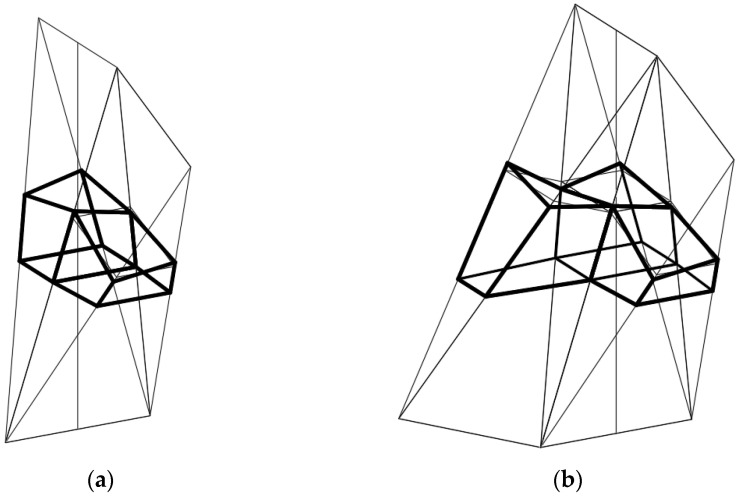
Subsequent meshes positioned in two orthogonal directions of the designed reference network *Γ*, the eaves network *B_v_*, and the complex structure *Σ*: (**a**) the first orthogonal direction, and (**b**) two orthogonal directions.

The location of the vertex *W_BC_*_12_ is determined so that it is contained in the plane (*y*, *z*), distant from *W_BC_*_11_ by the adopted value of d*v*_12_ and distant from the axis *u*_11_ by the height d*_BC_*_12_ of the triangle *W_BC_*_12_*W_CD_*_12_*W_AB_*_12_ measured from *W_BC_*_12_, where *W_BC_*_12_*W_CD_*_12_*W_AB_*_12_ should be congruent to *W_BC_*_11_*W_CD_*_11_*W_AB_*_11_. The above activities lead to the creation of the form presented in Figure 7a.

It is more convenient to control the shape of *Σ* when the proportions dd*_BC_*_12_ = d_*v*12_/d_*v*11_ and dd*_BC_*_12_ *=* d*_BC1_*_2_/d*_BC_*_11_ are used instead of the above-mentioned distances. *D_BC_*_12_ is the distance of *W_BC_*_12_ from *u*_12_. Similarly, *d_BC_*_11_ is the distance of *W_BC_*_11_ from *u*_11_. However, the distances can be calculated with the help of these coefficients. Adoption of the proportions between all meshes makes it possible to parametrize and control the shape of *Σ*, and, consequently, create diversified forms *Γ* and *Σ*. For the mesh *Γ*_12_ presented in Figure 7a, dd*_v_*_12_ = 1 and dd*_BC_*_12_ = 1. The parametrization should lead to a division of the forms *Σ* into different groups having similar geometric properties to predict the expected properties of the designed forms *Σ*. The subsequent tetrahedra *Γ*_1*j*_ (*j* = 1 to N, where N—the arbitrary natural number) belonging to the first orthogonal strip of *Γ* are constructed similar to *Γ*_12_.

All tetrahedra *Γ_i_*_1_ of the second orthogonal strip are formed in the same way as *Γ*_1*j*_ belonging to the first strip. The vertices *W_AD_*_21_, *W_BC_*_21_ and *W_AB_*_21_ of the first *Γ*_21_ tetrahedron, Figure 6, are assumed to be identical to the vertices of *W_AD_*_11_, *W_BC_*_11_ and *W_CD_*_11_ of *Γ*_11_, respectively, when creating the network *Γ*.

The fourth *W_CD_*_21_ vertex of *Γ*_21_ is constructed in the (*x*, *z*) plane in two arbitrary distances d_*u*21_ from *W_CD_*_11_ and d*_CD_*_21_ from *v*_11_. The method employed for parameterizing *Γ* consists in defining a certain number of coefficients expressing the proportions between the distances of the vertices of the subsequently created *Γ_ij_*’s axes. In the case of the *Γ*_21_ tetrahedron, these proportions can be given as follows: dd*_u_*_21_ = d*_u_*_21_/d*_u_*_11_ and dd*_CD_*_21_ = d*_CD_*_21_/d*_CD_*_11_. The above activities lead to the construction of the form presented in Figure 7b for which dd*_u_*_21_ = dd*_CD_*_21_ = 1.

**Figure 8:**
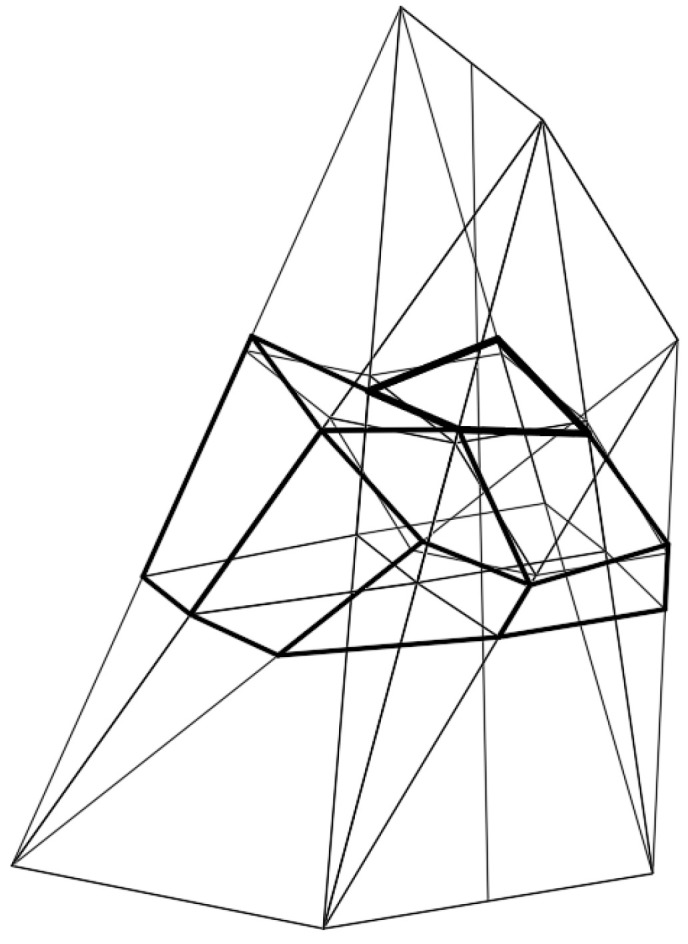
Four subsequent meshes of the designed reference network *Γ*, the eaves network *B_v_*, and the model *Σ*.

In the presented algorithm, there is no freedom in determining the vertices of *Γ*_22_ and other *Γ_ij_* tetrahedra arranged in diagonal strips (for *i* ≠ 1 or *j* ≠ 1). For the *Γ*_22_ tetrahedron, it is assumed that *W_AD_*_22_ = *W_BC_*_11_, *W_BC_*_22_ = *W_AD_*_12_, *W_CD_*_22_ = *W_CD_*_21_, *W_AB_*_22_ = *W_CD_*_11_. The above activities lead to the creation of the form presented in Figure 8.

Generally, for diagonal tetrahedra, *W_ADij_* = *W_BCi_*_−1*j*−1_, *W_BCij_* = *W_AD i_*_−1*j*_, *W_CDij_* = *W_CDij_*_−1_, *W_ABij_* = *W_CDi_*_−1*j*−1_. A slight modification of the described procedure allows one to set *W_ADij_*, *W_BCij_*, *W_CDij_* and *W_ABij_* at any points of the respective network side edges, not only at the vertices of the previously created tetrahedra. This reduction of the limitations in relation to the *Γ* network leads to fundamental changes in the proportions between the overall dimensions and the size of the elements of the building model shaped. The description of the modified procedure for shaping the *Γ* network goes beyond the scope of this work.

**Figure 9:**
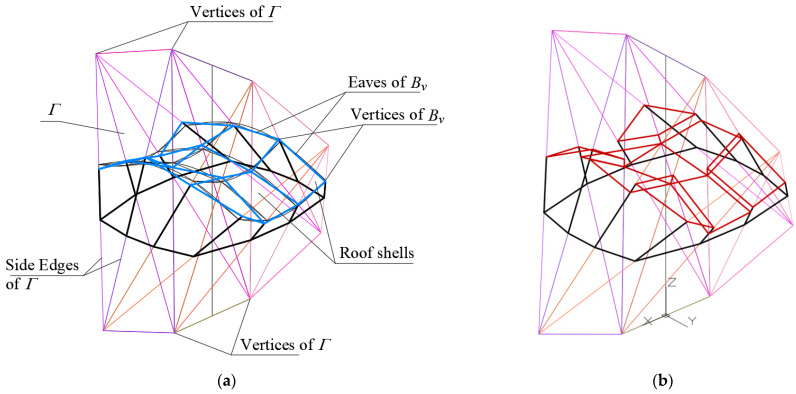
The designed reference network *Γ*, the eaves network *B_v_*, and the complex building structure *Σ* as well as their elements: (**a**) structure *Σ* roofed with continuous shell structure, (**b**) structure *Σ* roofed with discontinuous shell structure.

Each network *Γ*_1_ (Figure 8) formed according to the proposed algorithm is composed of four tetrahedra *Γ_ij_* (*i*, *j* = 1, 2) and can be extended to a symmetrical forms *Γ* (Figure 9a,b) for which (*x*, *z*) and (*y*, *z*) are the planes of symmetry. Thus, *Γ* consists of four symmetrical parts *Γ_i_* (*i* = 1 to 4). Based on the symmetrical reference network *Γ*, a polygonal *B_v_* network is created to define a multi-shell roof structure. *B_v_* is created so that some respective relationships between the location of the vertices of the roof structure and the vertices of *Γ* are adopted. The relationships are defined by means of the division coefficients of the pairs {*W_ABij_*, *W_ADij_*}, {*W_ABij_*, *W_BCij_*}, {*W_ADij_*, *W_CDij_*}, {*W_ADij_*, *W_BCij_*} of each *Γ_ij_* tetrahedron by the vertices of the eaves polygonal network *B_v_*, reference surface and the base of the form *Σ*.

The first group {dd*_SAij_*, dd*_SBij_*, dd*_SCij_* and dd*_SDij_*} of the division coefficients is used to determine the points *S_Aij_*, *S_Bij_*, *S_Cij_* and *S_Dij_* on the side edges of *Γ* defining the reference surface *ω_r_* to search for the network *B_v_*. The second group {dd*_Aij_*, dd*_Bij_*, dd*_Cij_* and dd*_Dij_*} of the division coefficients is used to determine the points *A_ij_*, *B_ij_*, *C_ij_* and *D_ij_* constituting the vertices of the polygonal network *B_v_*. For the networks under consideration: (a) all acceptable values of the above-mentioned two types of the coefficients are in the range (0, 1), (b) the reference surfaces defined by the points *S_Aij_*, S*_Bij_*, S*_Cij_* and S*_Dij_* have negative Gaussian curvature. These limitations results from the geometric properties of the network *Γ* made. They proves the innovative nature of the performed analysis and the proposed procedure. In the case of the topics discussed so far in other articles, the values of these coefficients are within the range (1, +∞), and the reference surface is characterized by the positive Gaussian curvature. The case in which the values of the above-mentioned coefficients are within the range (−∞, 0) is similar to that with the range (1, +∞) but requires some modifications.

As it is convenient to define the positions of the vertices *A_ij_*, *B_ij_*, *C_ij_* and *D_ij_* with respect to the reference surface *ω_r_*, it is rational to use coefficients taking account of the difference in the levels of the vertices belonging to *ω_r_* and *B_v_*. For this purpose, the quotient of: (a) the respective values of the division coefficients of pairs {*W_ABij_*, *W_ADij_*}, {*W_ABij_*, *W_BCij_*}, {*W_ADij_*, *W_CDij_*} and {*W_ADij_*, *W_BCij_*} by: (a) points *S_Aij_*, *S_Bij_*, *S_Cij_* and *S_Dij_*, and (b) points *A_ij_*, *B_ij_*, *C_ij_* and *D_ij_* can be calculated. However, the exact description of this problem is presented by Abramczyk [24] and goes beyond the scope of the present article.

In the considered examples, the values of the partition coefficients used for the meshes *B_vij_* lead to small or big folding of the examined roof structure covered with complete transformed shells separated by ribs. On the other hand, the base of the modeled building is flat and horizontal, so the *z*-coordinates of all base points, belonging to the *P_b_* plane, are the same. The arbitrary level of the *P_b_* plane is adopted as constant *z_P_* from the interval <0, dd*_uv_*_11_>. The coordinates of all points *P_Aij_*, *P_Bij_*, *P_Cij_* and *P_Dij_* of the base belonging to the edges of the facade walls are obtained as a result of the intersection of the plane *P_b_* with all *Γ*’s side edges.

The proportions taken into account, inter alia, the length, width and height of the entire *Σ* model and its fragments depend on the mutual position of the vertices of the reference polyhedral *Γ* network, the vertices of the polygonal *B_v_* network, the base plane *P_b_* and the reference surface used. These proportions are determined by the assumed values of the independent variables and the relationships between the values of the dependent and independent variables. In order to illustrate the impact of adopting different sets of values of the above-mentioned variables on the form of the *Γ* model, three examples of complex folded building forms covered with different shell roof structures characterized by the negative Gaussian curvature are presented in the next section.

## 5. Results

There are presented three examples showing the way of using the developed procedure in the process of shaping various unconventional complex forms of buildings, including the possibility of parameterizing these forms. In order to create the first tetrahedron *Γ*_11_ of a polyhedral network *Γ*, the values of the following parameters have to be adopted: (1) the distances d*_v_*_11_ of two vertices *W_BC_*_11_ and *W_AD_*_11_ belonging to the first axis *v*_11_ (Figure 6), (2) the ratio dd*_uv_*_11_ of the above distance d*_uv_*_11_ to the distance d*_v_*_11_ between the oblique axes *u*_11_ and *v*_11,_ (3) the ratio dd*_u_*_11_ of the distance d*_u_*_11_ of two vertices *W_AB_*_12_ and *W_CD_*_12_ belonging to the second axis *u*_11_ to d*_v_*_11_. The values employed are given in Table 1.

Identical values are adopted for the remaining congruent tetrahedra located in the orthogonal directions of the developed *Γ*_1_ reference network. As a result of the respective composing of *Γ*_11_, *Γ*_21_ and *Γ*_12_ a network *Γ*_1_ can be created. The vertices of *Γ*_22_ are lain at four vertices of the above tetrahedrons obtained previously. The co-ordinates of these vertices are given in Table A1 in Appendix.

Then, to determine the positions of the vertices *S_A_*_11_, *S_B_*_11_, *S_C_*_11_ and *S_D_*_11_ of the first mesh of the reference surface *ω_r_* lying in the side edges of *Γ*_11_, the coefficients dd*_SA_*_11_, dd*_SB_*_11_, dd*_SC_*_11_ and dd*_SD_*_11_ constituting the division coefficients of the vertices of the *Γ*_11_ tetrahedron by *S_A_*_11_, *S_B_*_11_, *S_C_*_11_ and *S_D_*_11_ should be adopted. The arbitrary values of these coefficients are presented in Table A2 in the Appendix. The calculated values of the coordinates of these vertices are presented in Table A3 in the Appendix.

**Figure 10:**
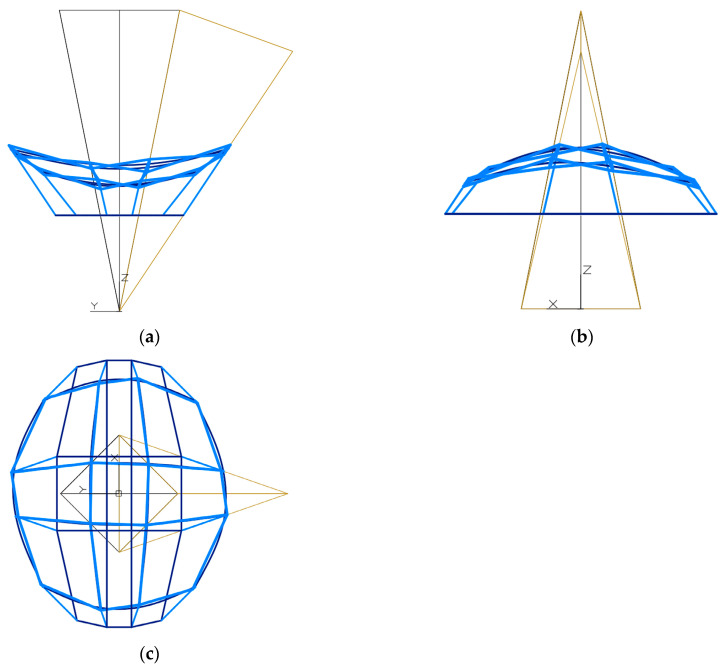
The designed reference network *Γ*, the first eaves network *B_v_*, and the first complex building structure *Σ*: (**a**) the front view, (**b**) the back view, (**c**) the top view.

Another operation provided for in the algorithm of the procedure is to determine the positions of the vertices *A*_11_, *B*_11_, *C*_11_ and *D*_11_ of the first mesh *B_v_*_11_ of the polygonal net *B_v_*_1_. For the form shaped (Figure 10) the appropriate values of the coefficients dd*_A_*_11_, dd*_B_*_11_, dd*_C_*_11_ and dd*_D_*_11_ of the vertices of the *Γ*_11_ mesh by *A*_11_, *B*_11_, *C*_11_ and *D*_11_ are adopted. These values are given in Table A4 in Appendix. The *p_z_* level of the base plane of the considered form is equal to 15,920 mm in [*x*,*y*,*z*]. The individual vertices *P_A_*_11_, *P_B_*_11_, *P_C_*_11_ and *P_D_*_11_ of this base can be constructed as the points of intersection of the horizontal plane *p_z_* with the side edges of *Γ*_1_.

The following meshes of the *Γ*_1_, *B_v_*_1_ nets and the reference surface are created in the same way. Alternatively, some vertices must be adopted at the positions of the corresponding vertices belonging to the previously constructed meshes.

A characteristic feature of the folded forms created by the procedure is the different inclination of the adjacent facade walls to the vertical. The form presented in Figure 10a–c is characterized by two opposite façade walls directed in the *x*-axis direction and inclined with their bases inwards, while the other two façade walls are inclined with their bases outwards.

On the other hand, a specific feature distinguishing this form from two following forms is the fact that its elevation walls with their bases inwards are much longer than two other elevation walls with bases inclined outwards. As a result, the form has an elongated elliptical shape when projected onto a horizontal plane, and the *x* axis can be considered as the principal axis of the imaginary ellipse (Figure 10c).

Three exemplary forms *Σ* were created based on the same reference network *Γ*. For these forms, appropriate and different values of three sets of the partition coefficients related to the points of the created reference surfaces, eaves networks and the bases were adopted. The first set was adopted so that the form *Σ* has an elongated form in the direction of the axis *x* in the projection on the plane (*x*, *y*) and the base plane is closer to the lower vertices of the reference network *Γ* (Figure 10).

**Figure 11:**
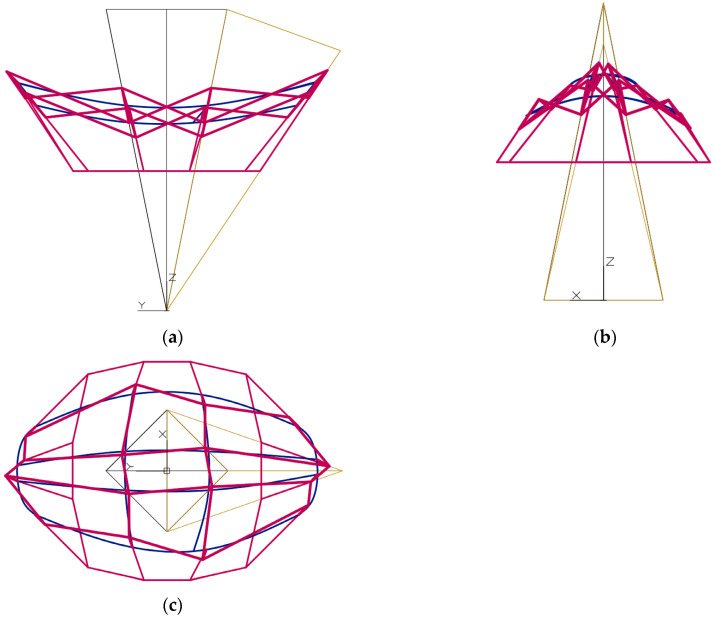
The designed reference network *Γ*, the second eaves network *B_v_*, and the second complex building structure *Σ*: (**a**) the front view, (**b**) the back view, (**c**) the top view.

**Figure 12:**
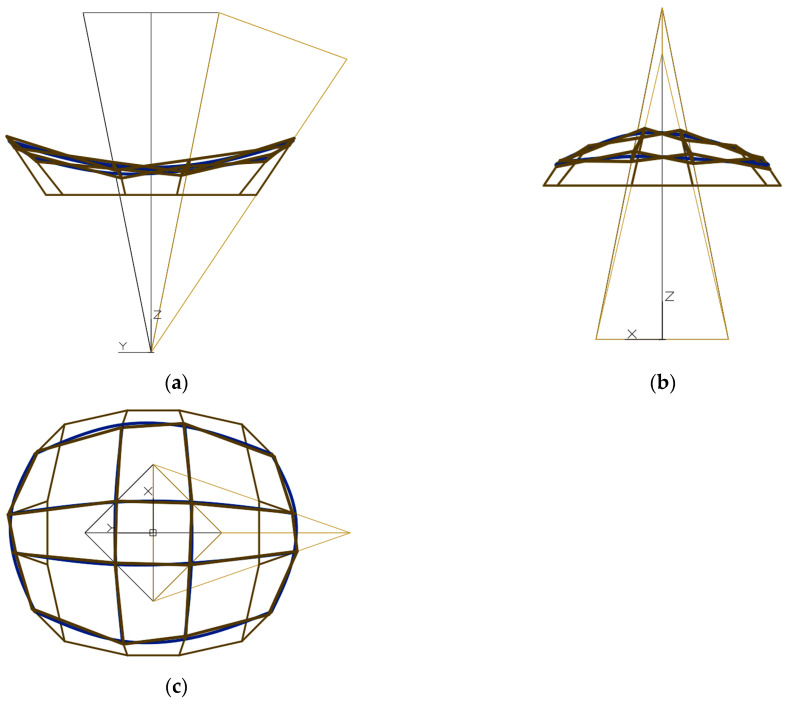
The designed reference network *Γ*, the third eaves network *B_v_*, and the third complex building structure *Σ*: (**a**) the front view, (**b**) the back view, (**c**) the top view.

The second set of the partition coefficients was adopted so that the second form *Σ* has an elongated form in the direction of the *y* axis in the projection on the (*x*, *y*) plane, the plane of the base is halfway between the upper and lower vertices of the network *Γ*, and the points of the reference surface *ω_r_* lie close to the base (Figure 11). The third set of the partition coefficients was adopted so that the third form *Σ* has a square form when projected onto the (*x*, *y*) plane, the base plane is halfway between the upper and lower vertices of the network *Γ*, and the points of the reference surface *ω_r_* lie further from the base than in the previous case (Figure 12). The differences between the essential dimensions of the above forms are noticeable when comparing the respective views of each of the above-mentioned three forms.

The values of the relevant parameters and coordinates of these forms are given in Table A2, Table A3, Table A4, Table A5, Table A6, Table A7, Table A8, Table A9 and Table A10. It is worth noticing that some geometric properties of two new forms, distinguishing them from the previously presented form are as follows. Two elevation walls of the form shown in Figure 11, whose direction is in line with the *y*-axis and the bases are tilted outwards, are much longer than the elevation walls titled with their bases inwards and running along the *x*-axis. The *p_z_* level of the base plane of the considered form is equal to 31,010 mm in [*x*,*y*,*z*].

On the other hand, all façade walls of the form shown in Figure 12 are of similar length regardless of whether their bases are tilted outwards or inwards. As a result, the projection of the third form onto a horizontal plane has a shape similar to a circle or a square. The differentiation of three above-mentioned general forms is generated by the deliberate differentiation of the values of the single division coefficients used. A discussion on this subject is presented in the next section. The *p_z_* level of the base plane of the considered form is equal to 23,194 mm in [*x*,*y*,*z*].

## 6. Discussion

The following relationships can be noticed from the observation of geometric properties of the orthogonal projections of three different structures presented in the previous section.

The inclination of the facade walls with their bases towards the inside or outside of a considered building form depends on the orthogonal directions of two types of axes *v_ij_* and *u_ij_* of a polyhedral reference network *Γ*. For example, such a network is presented in Figure 10, Figure 11 and Figure 12. If the considered dimension of the building form is consistent with the axis *v*_11_ of the *Γ* network, located above the base (*x*, *y*) of the form, as can be seen in Figure 10a, Figure 11a, Figure 12a the projections show facade walls inclined towards the inside of the form. However, if the dimension of the form is considered orthogonal to the axis *v*_11_ located above the base of the building, as it is shown in Figure 10b, Figure 11b, Figure 12b, then the elevation walls tilted with their bases outside the considered form are visible.

The overall dimensions of a building form in all three directions of the axes of its local system [*x*,*y*,*z*] are determined by the shape of the base edge line, the eaves line and their mutual position in the horizontal and vertical directions (Figure 10, Figure 11 and Figure 12).

The locations of the edge lines of the base and the eaves of the created form depends on the position of their vertices in the side edges of the polyhedral reference network *Γ*. The more the levels of the eaves line vertices are closer to the level of the axis *u*_11_, the more the base is stretched along the axis *x* and compressed along the axis *y*. However, in order to obtain similar dimensions of the form (dimensions of the edge lines of the base and the eaves) in two orthogonal horizontal directions, the vertices of the eaves edge and the vertices of the base edge should be placed at levels equidistant (or close to such) from the level of the horizontal plane passing through the midpoint of the straight line normal to *u*_11_ and *v*_11_, on both sides of this middle plane. All levels are defined by horizontal planes and their distances from the principal plane (*x*, *y*).

The shape of the eaves line *B_v_* of a building form may be generated by the division coefficients of the respective pairs of vertices belonging to the polyhedral reference network *Γ* by the vertices of *B_v_*. On the other hand, the shape of the base edge line can be determined by the coefficient defining their plane level. Control of the mutual position of the eaves line *B_v_* and the base edges can be done by double division coefficients of the vertices of *Γ* by the eaves vertices and the base vertices. This issue goes beyond the scope of the paper.

To obtain an elongated horizontal projection of a building base along the axis *x*, the base level coefficient dd*_P_* should be taken significantly lower than 0.5 (Figure 10). To generate a horizontal projection of the building base elongated along the axis *y*, the coefficient dd*_P_* should be assumed significantly greater than 0.5. However, in order to obtain a horizontal projection of the building base with a comparable length of two overall dimensions measured in the *x* and *y* directions, the dd*_P_* coefficient should be adopted close to 0.5.

If the examined polyhedral reference network *Γ* has properties analogous to the networks presented in Figure 11 and Figure 12, i.e., *u*_11_ is identical to *x* of [*x*,*y*,*z*], *v*_11_ is perpendicular to *u*_11_ and distant by d*_uv_*_11_ from *z*, then the following are true. To obtain an elongated plan view of the designed eaves line along the axis *x*, the coefficients dd*_Aij_*, dd*_Bij_*, dd*_Cij_* and dd*_Dij_* should be taken significantly less than 0.5. To obtain an elongated plan view of the eaves line along the axis *y*, the coefficients dd*_Aij_*, dd*_Bij_*, dd*_Cij_* and dd*_Dij_* should be significantly greater than 0.5. To generate a horizontal projection of roof eaves line characterized by comparable overall dimensions measured in the *x* and *y* directions, the arbitrary division coefficients close to 0.5 should be taken.

In order to generate an innovative form of a building having two façade walls inclined inwards and another two outwards of the form, and being close to a circle when projected onto a horizontal plane, the absolute values of the differences dd*_Aij_*—0.5, dd*_Bij_*—0.5, dd*_Cij_*—0.5 and dd*_Dij_*—0.5 should be adopted equal or close to an absolute value of the difference dd*_P_*—0.5, especially for all central meshes of the net *Γ*.

The examined general forms are to be influenced by corrugation of the polygonal eaves network Bv generated by the size of the differences between: (a) the division coefficients of the vertices of polyhedral reference network *Γ* by the vertices *A_ij_*, *B_ij_*, *C_ij_* and *D_ij_* of *B_v_*, and (b) the division coefficients of the same vertices of *Γ* by the corresponding points *S_Aij_*, *S_Bij_*, *S_Cij_* and *S_Dij_* of a reference surface. In addition, these forms are to be influenced by the ratio between the size of this corrugation and the height of the entire form, which depends on the division coefficients of the vertices of the polyhedral reference network by points belonging to three groups: (a) base vertex group, (b) reference surface point group, and (c) eaves vertex group. The discussion about the influence of some important proportions between the values of the above-mentioned coefficients and the geometrical properties of the building forms shaped goes beyond the scope of the article and requires definition of the so-called multiple division coefficients.

## 7. Conclusions

An innovative procedure significantly extending the method for modeling unconventional building forms with complex folded elevations and multi-segment transformed shell roof structure was developed. The specific features of the elaborated procedure is are: (a) an arrangement of many complete transformed shells of a roof structure in the three-dimensional space in accordance with the properties of a regular saddle surface with negative Gaussian curvature, (b) achievement of different inclinations of elevation walls to the vertical, including outside and inside of the same building. Thus, the final innovative method presented in the article fills the important gap existing in the current knowledge.

The algorithm of the procedure was presented on three specific examples of creating complex building forms using appropriate proportions between the distances of all characteristic vertices of their elevation walls and roofs. The procedure enables the designer to take some dependencies determining the respective interrelationships between the distances of the subsequent vertices of the designed reference network deciding on the complex shape of elevation walls. On the basis of the created reference network, the vertices of the polygonal eaves network determining the arrangement and forms of all multi-segment shells of the determined roof structures are defined by means of the division coefficients. The ranges of variability of the above coefficients are also defined so that it is possible to create a few specific types of the building forms under consideration.

A characteristic feature of the presented procedure is that the vertices of each polyhedral reference network are divided into two groups such that the vertices belonging to these groups define two families of the orthogonal axes lying on both sides of the reference surface employed. This leads to a complex shell roof structure the overall shape of which is characterized by the negative Gaussian curvature. This property of the designed roof form is generated by a model whose division coefficients of the vertices belonging to the reference polyhedral network by the roof eaves vertices are in the range (0, 1).

There is a need for searching for further unconventional forms of roofs and elevations as well as their innovative structural systems supporting the transformed folded shell roof sheeting. Some extend laboratory tests in the field of thin-walled folded sheeting transformed elastically into various forms of saddle surfaces are also going to be performed.

## Figures and Tables

**Table 1:** The values of the initial parameters of *Γ*_1_.

Initial Parameter	d*_v_*_11_ [mm]	dd*_uv_*_11_	dd*_u_*_11_
	20,000	5	1

## Nomenclature

*Γ*	the polyhedral reference network
*B_v_*	the polygonal eaves network
*Ω*	the complex shell roof structure
*Σ*	the whole complex free form building structure
*ω_r_*	the reference regular surface
*P_b_*	the horizontal base plane
*Ω_ij_*	the individual mesh of *Ω*
*Γ_ij_*	the individual mesh of *Γ*
*W_ABij_, W_ADij_*	the vertices of *Γ_ij_*
*B_vij_*	the single mesh of *B_v_*
*A_ij_, B_ij_,*	the vertices of *B_vij_*
*S_Aij_, S_Bij_*	the points of *ω_r_*
*u_ij_, v_ij_*	the axes of *Γ_ij_*
*a_ij_, b_ij_*	the side edges of *Γ_ij_*
dd*_SAij_,* dd*_SBij_*	division coefficients related to the points of *ω_r_*
dd*_Aij_,* dd*_Bij_*	division coefficients related to the points of *B_v_*
[*x*,*y*,*z*]	the orthogonal coordinate system
(*x, y*)	principal plane of [*x*,*y*,*z*]

## Appendix A

**Table A1:** The coordinates of the vertices *W_ABij_*, *W_CDij_*, *W_ADij_*, *W_BCij_* (for *i*, *j* = 1, 2) of the basic polyhedral reference network *Γ*_1_.

Point	*x*-Coordinate [mm]	*y*-Coordinate [mm]	*z*-Coordinate [mm]
*W_AB_*_11_	−10,000	0	0
*W_CD_*_11_	10,000	0	0
*W_AD_*_11_	0	10,000	50,000
*W_BC_*_11_	0	−10,000	50,000
*W_AB_*_12_	−10,000	0	0
*W_CD_*_12_	28,793	0	6840
*W_AD_*_12_	0	10,000	50,000
*W_BC_*_12_	0	−10,000	50,000
*W_AB_*_21_	0	−28,793	43,160
*W_CD_*_21_	0	−10,000	50,000
*W_AD_*_21_	−10,000	0	0
*W_BC_*_21_	10,000	0	0
*W_AB_*_22_	28,793	0	6840
*W_CD_*_22_	10,000	0	0
*W_AD_*_22_	0	−28,793	43,160
*W_BC_*_21_	0	−10,000	50,000

**Table A2:** The initial division coefficients defining the positions of the points *SA_ij_*, *SB_ij_*, *SC_ij_*, *SD_ij_* and the *B_v_*’s vertices of the first structure *Σ*_1_ for *i*, *j* = 1, 2.

Ratio	Value
dd*_SA_*_11_	0.465
dd*_SB_*_11_	0.478
dd*_SC_*_11_	0.489
dd*_SD_*_11_	0.489
dd*_SA_*_12_	0.478
dd*_SB_*_12_	0.600
dd*_SC_*_12_	0.617
dd*_SD_*_12_	0.489
dd*_SA_*_22_	0.327
dd*_SB_*_22_	0.450
dd*_SC_*_22_	0.600
dd*_SD_*_22_	0.478
dd*_A_*_11_	0.480
dd*_B_*_11_	0.462
dd*_C_*_11_	0.500
dd*_D_*_11_	0.474
dd*_A_*_12_	0.462
dd*_B_*_12_	0.600
dd*_C_*_12_	0.600
dd*_D_*_12_	0.500
dd*_A_*_22_	0.343
dd*_B_*_22_	0.435
dd*_C_*_22_	0.600
dd*_D_*_22_	0.462

**Table A3:** The coordinates of the points defining the reference surface *ω*_1_ of the first structure *Σ*_1_.

Point	*x*-Coordinate [mm]	*y*-Coordinate [mm]	*z*-Coordinate [mm]
*S_A_*_11_	−5354.1	4645.9	23,228.7
*S_B_*_11_	−5221.6	−4778.5	23,892.5
*S_C_*_11_	5107.3	−4892.7	24,463.6
*S_D_*_11_	5107.3	4892.7	24,463.6
*S_A_*_12_	−5221.6	−4778.5	23,892.5
*S_B_*_12_	−3748.6	−18,000.2	26,980.8
*S_C_*_12_	3826.3	−17,776.5	26,645.6
*S_D_*_12_	5107.3	−4892.7	24,463.6
*S_A_*_21_	−19,413.7	3257.6	20,899.9
*S_B_*_21_	−19,366.8	−3273.9	20,970.3
*S_C_*_21_	−5221.6	−4778.5	23,892.5
*S_D_*_21_	−5354.1	4645.9	23,228.7
*S_A_*_22_	−19,366.8	−3273.9	20,970.3
*S_B_*_22_	−15,836.3	−12,957.2	23,184.0
*S_C_*_22_	−3748.6	−18,000.2	26,980.8
*S_D_*_22_	−5221.6	−4778.5	23,892

**Table A4:** The coordinates of the vertices *A_ij_*, *B_ij_*, *C_ij_*, *D_ij_* (for *i*, *j* = 1, 2) of the eaves edge net *B_v_*_1_ (and the first structure *Σ*_1_).

Point	*x*-Coordinate [mm]	*y*-Coordinate [mm]	*z*-Coordinate [mm]
*A*_11_	−5200.3	4799.7	23,998.5
*B*_11_	−5375.4	−4624.6	23,122.8
*C*_11_	5000	−5000	25,000
*D*_11_	5261.2	4738.8	23,693.8
*A*_12_	*xB*_11_	*yB*_11_	*zB*_11_
*B*_12_	−3748.6	−18,000.2	26,980.8
*C*_12_	4002.5	−17,2693	25,885.2
*D*_12_	*xC*_11_	*yC*_11_	*zC*_11_
*A*_21_	−19,849.7	3106.2	20,246.4
*B*_21_	−18,930.8	−3425.3	21,623.8
*C*_21_	*xB*_11_	*yB*_11_	*zB*_11_
*D*_21_	*xA*_11_	*yA*_11_	*zA*_11_
*A*_22_	*xB*_21_	*yB*_21_	*zB*_21_
*B*_22_	−16,258.5	−12,535.1	22,651.6
*C*_22_	*xB*_12_	*yB*_12_	*zB*_12_
*D*_22_	*xB*_11_	*yB*_11_	*zB*_11_

**Table A5:** The initial division coefficients defining the positions of the points *S_Aij_*, *S_Bij_*, *S_Cij_*, *S_Dij_* and the *B_v_*’s vertices of the second structure *Σ*_2_ for *i*, *j* = 1, 2.

Ratio	Value
dd*_SA_*_11_	0.682
dd*_SB_*_11_	0.682
dd*_SC_*_11_	0.682
dd*_SD_*_11_	0.682
dd*_SA_*_12_	0.682
dd*_SB_*_12_	0.869
dd*_SC_*_12_	0.882
dd*_SD_*_12_	0.682
dd*_SA_*_22_	0.564
dd*_SB_*_22_	0.750
dd*_SC_*_22_	0.869
dd*_SD_*_22_	0.682
dd*_A_*_11_	0.624
dd*_B_*_11_	0.739
dd*_C_*_11_	0.624
dd*_D_*_11_	0.739
dd*_A_*_12_	0.739
dd*_B_*_12_	0.819
dd*_C_*_12_	0.919
dd*_D_*_12_	0.624
dd*_A_*_22_	0.510
dd*_B_*_22_	0.805
dd*_C_*_22_	0.819
dd*_D_*_22_	0.739

**Table A6:** The coordinates of the points defining the reference surface *ω*_2_ of the second structure *Σ*_2_.

Point	*x*-Coordinate [mm]	*y*-Coordinate [mm]	*z*-Coordinate [mm]
*S_A_*_11_	−3184	6816	34,080
*S_B_*_11_	−3184	−6816	34,080
*S_C_*_11_	3184	−6816	34,080
*S_D_*_11_	3184	6816	34,080
*S_A_*_12_	−3184	−6816	34,080
*S_B_*_12_	−1814.9	−24,600.6	37,870.4
*S_C_*_12_	1868.1	−24,539.6	37,793.5
*S_D_*_12_	3184	−6816	34,080
*S_A_*_21_	−12,549.5	56,415	31,189
*S_B_*_21_	−12,546.6	−5642.5	31,193.4
*S_C_*_21_	−3184	−6816	34,080
*S_D_*_21_	−3184	6816	34,080
*S_A_*_22_	−12,546.6	−5642.5	31,193.4
*S_B_*_22_	−71,984	−21,595.4	34,080
*S_C_*_22_	−1814.9	−24,600.6	37,870.4
*S_D_*_22_	−3184	−6816	34,080

**Table A7:** The coordinates of the vertices *A_ij_*, *B_ij_*, *C_ij_*, *D_ij_* (for *i*, *j* = 1, 2) of the eaves edge net *B_v_*_1_ (and the second structure *Σ*_2_).

Point	*x*-Coordinate [mm]	*y*-Coordinate [mm]	*z*-Coordinate [mm]
*A*_11_	−3761.4	6238.6	31,193
*B*_11_	−2606.7	−7393.3	36,966.7
*C*_11_	3761.4	−6238.6	31,193
*D*_11_	2606.7	7393.3	36,966.7
*A*_12_	*xB*_11_	*yB*_11_	*zB*_11_
*B*_12_	−1811.7	−23,577.4	35,340.5
*C*_12_	757.6	−26,612.6	39,890
*D*_12_	*xC*_11_	*yC*_11_	*zC*_11_
*A*_21_	−914.7	6209.3	33,639.4
*B*_21_	−14,114.4	−5098	28,843.3
*C*_21_	*xB*_11_	*yB*_11_	*zB*_11_
*D*_21_	*xA*_11_	*yA*_11_	*zB*_11_
*A*_22_	*xB*_21_	*yB*_21_	*zB*_21_
*B*_22_	−1811.7	−23,577.4	35,340.5
*C*_22_	*xB*_12_	*yB*_12_	*zB*_12_
*D*_22_	*xB*_11_	*yB*_11_	*zB*_11_

**Table A8:** The initial division coefficients defining the positions of the points *S_Aij_*, *S_Bij_*, *S_Cij_*, *S_Dij_* and the *B_v_*’s vertices of the third structure *Σ*_3_ for *i*, *j* = 1, 2.

Ratio	Value
dd*_SA_*_11_	0.544
dd*_SB_*_11_	0.544
dd*_SC_*_11_	0.552
dd*_SD_*_11_	0.552
dd*_SA_*_12_	0.544
dd*_SB_*_12_	0.715
dd*_SC_*_12_	0.721
dd*_SD_*_12_	0.552
dd*_SA_*_22_	0.460
dd*_SB_*_22_	0.600
dd*_SC_*_22_	0.715
dd*_SD_*_22_	0.544
dd*_A_*_11_	0.545
dd*_B_*_11_	0.531
dd*_C_*_11_	0.567
dd*_D_*_11_	0.536
dd*_A_*_12_	0.531
dd*_B_*_12_	0.730
dd*_C_*_12_	0.706
dd*_D_*_12_	0.567
dd*_A_*_22_	0.475
dd*_B_*_22_	0.585
dd*_C_*_22_	0.730
dd*_D_*_22_	0.531

**Table A9:** The coordinates of the points defining the reference surface *ω*_3_ of the third structure *Σ*_3_.

Point	*x*-Coordinate [mm]	*y*-Coordinate [mm]	*z*-Coordinate [mm]
*S_A_*_11_	−4549.4	5450.6	27,252.8
*S_B_*_11_	−4416.7	−5577.5	27,228.5
*S_C_*_11_	4484.2	−5515.9	27,577.8
*S_D_*_11_	4484.2	5515.9	27,577.8
*S_A_*_12_	−4416.7	−5577.5	27,228.5
*S_B_*_12_	−2847.1	−20,596	30,871.7
*S_C_*_12_	2790.6	−20,758.7	31,115.5
*S_D_*_12_	4484.2	−5515.9	27,577.8
*S_A_*_21_	−15,814.4	45,076	26,295.0
*S_B_*_21_	−15,544.2	−4601.5	26,700.1
*S_C_*_21_	−4416.7	−5577.5	27,228.5
*S_D_*_21_	−4549.4	5450.6	27,252.8
*S_A_*_22_	−15,544.2	−4601.5	26,700.1
*S_B_*_22_	−11,517.3	−17,276.3	28,631.9
*S_C_*_22_	−2847.1	−20,596	30,871.7
*S_D_*_22_	4484.2	−5515.9	27,577.8

**Table A10:** The coordinates of the vertices *A_ij_*, *B_ij_*, *C_ij_*, *D_ij_* (for *i*, *j* = 1, 2) of the eaves edge net *B_v_*_1_ (and the third structure *Σ*_3_).

Point	*x*-Coordinate [mm]	*y*-Coordinate [mm]	*z*-Coordinate [mm]
*A*_11_	−4401.7	5598.3	27,991.6
*B*_11_	−4694.9	−5305.1	26,525.3
*C*_11_	4330.5	−5669.5	28,347.7
*D*_11_	4638.4	5361.6	26,808.1
*A*_12_	*xB*_11_	*yB*_11_	*zB*_11_
*B*_12_	−2695.7	−21,031.9	31,525.1
*C*_12_	2942	−20,322.7	30,462.1
*D*_12_	*xC*_11_	*yC*_11_	*zC*_11_
*A*_21_	−16,250.4	4356.2	25,641.5
*B*_21_	−15,101.8	−4743.8	27,344.6
*C*_21_	*xB*_11_	*yB*_11_	*zB*_11_
*D*_21_	*xA*_11_	*yA*_11_	*zA*_11_
*A*_22_	*xB*_21_	*yB*_21_	*zB*_21_
*B*_22_	−11,939.5	−16,854.1	28,009.4
*C*_22_	*xB*_12_	*yB*_12_	*zB*_12_
*D*_22_	*xB*_11_	*yB*_11_	*zB*_11_

## References

[1] Yu W.W., LaBoube R.A., Chen H. (2000). Cold Formed Steel Design.

[2] Abramczyk J. (2017). Shell Free Forms of Buildings Roofed with Transformed Corrugated Sheeting, Monograph.

[3] Reichhart A. (2002). Geometrical and Structural Shaping Building Shells Made up of Transformed Flat Folded Sheets.

[4] Abramczyk J. (2021). Folded Sheets as a Universal Material for Shaping Transformed Shell Roofs. Materials.

[5] Grey A. (1999). Modern Differential Geometry of Curves and Surfaces with Mathematica.

[6] Abramczyk J. (2021). Transformed Shell Structures Determined by Regular Networks as a Complex Material for Roofing. Materials.

[7] Zhang L., Bhatti M.M., Michaelides E.E., Marin M., Ellahi R. (2022). Hybrid nanofluid flow towards an elastic surface with tantalum and nickel nanoparticles, under the influence of an induced magnetic field. Eur. Phys. J. Spec. Top..

[8] Abouelregal A.E., Marin M. (2020). The response of nanobeams with temperature-dependent properties using state-space method via modified couple stress theory. Symmetry.

[9] Abramczyk J. (2020). Symmetric Free Form Building Structures Arranged Regularly on Smooth Surfaces with Polyhedral Nets. Symmetry.

[10] Abel J.F., Mungan I. (2011). Fifty Years of Progress for Shell and Spatial Structures.

[11] Saitoh M. (2001). Recent Spatial Structures in Japan. J. JASS.

[12] Davis J.M., Bryan E.R. (1982). Manual of Stressed Skin Diaphragm Design.

[13] Abramczyk J. (2019). Symmetric Shape Transformations of Folded Shell Roofs Determining Creative and Rational Shaping of Building Free Forms. Symmetry.

[14] Reichhart A. Principles of designing shells of profiled steel sheets. Proceedings of the X International Conference on Lightweight Structures in Civil Engineering.

[15] Biswas M., Iffland J.S.B. Metal decks used to form hypar-shell panels. Proceedings of the 2nd Speciality Conference on Cold-Formed Steel Structures.

[16] Prokopska A., Abramczyk J. (2019). Responsive Parametric Building Free Forms Determined by Their Elastically Transformed Steel Shell Roofs. Buildings.

[17] Abramczyk J. (2020). Innovative Building Forms Determined by Orthotropic Properties of Folded Sheets Transformed into Roof Shells. J. JASS.

[18] Pottmann H., Asperi A., Kilian A., Hofer M. (2007). Architectural Geometry.

[19] Attard M.M. (1986). Nonlinear theory of Non-Uniform Torsion of Thin-Walled Open Beams. Thin-Walled Struct..

[20] Vlasov V.Z. (1959). Tonkostennye Uprugie Sterzhni.

[21] Vaziri A. (2009). Mechanics of highly deformed elastic shells. Thin-Walled Struct..

[22] Samyn P.E. Structures isobarres et isonoeuds. Proceedings of the 2nd International Conference on Space Structures—University of Surrey.

[23] Reichhart A. Corrugated Deformed Steel Sheets as Material for Shells. Proceedings of the International Conference of Lightweight Structures in Civil Engineering.

[24] Abramczyk J. (2021). Transformed Corrugated Shell Units Used as a Material Determining Unconventional Forms of Complex Building Structures. Materials.

[25] Prokopska A., Abramczyk J. (2017). Innovative systems of corrugated shells rationalizing the design and erection processes for free building forms. Archit. Civ. Eng. Environ..

[26] Sharma A. (2015). Urban greenways: Operationalizing design syntax and integrating mathematics and science in design. Front. Archit. Res..

[27] Hasgül E. Space as configuration: Patterns of space and culture. Proceedings of the ARCHTHEO 2015_9th Conference: Theory and History of Architecture.

[28] Eekhout M. Form as a Bridge between Architectural, Structural and Industrial Design. Proceedings of the 4th International Colloqium on Structural Morphology IASS: Spatial Lattice and Tension Structures.

[29] Craciun E.M., Marin M., Pop N. (2020). Some Results in Green–Lindsay Thermoelasticity of Bodies with Dipolar Structure. Mathematics.

[30] Abbas I., Marin M., Saeed T. (2020). A GL model on thermo-elastic interaction in a poroelastic material using finite element method. Symmetry.

[31] Gronostajska B.E., Tarczewski R., Jablonska J. (2021). Architecture, City, People and Structure. Buildings.

[32] Rębielak J. Architectonic forms and engineering systems designed by application of method of superposition. Proceedings of the 7th International Conference on Structural Engineering, Mechanics and Computation.

